# Multiscale brain modeling: bridging microscopic and macroscopic brain dynamics for clinical and technological applications

**DOI:** 10.3389/fncel.2025.1537462

**Published:** 2025-02-19

**Authors:** Ondrej Krejcar, Hamidreza Namazi

**Affiliations:** ^1^Skoda Auto University, Na Karmel, Mlada Boleslav, Czechia; ^2^Media and Games Center of Excellence, Universiti Teknologi Malaysia, Johor Bahru, Malaysia; ^3^School of Engineering, Monash University, Selangor, Malaysia

**Keywords:** multiscale brain modeling, computational neuroscience, big data, AI, neuroimaging, clinical applications, brain function, multi-disciplinary research

## Abstract

The brain’s complex organization spans from molecular-level processes within neurons to large-scale networks, making it essential to understand this multiscale structure to uncover brain functions and address neurological disorders. Multiscale brain modeling has emerged as a transformative approach, integrating computational models, advanced imaging, and big data to bridge these levels of organization. This review explores the challenges and opportunities in linking microscopic phenomena to macroscopic brain functions, emphasizing the methodologies driving progress in the field. It also highlights the clinical potential of multiscale models, including their role in advancing artificial intelligence (AI) applications and improving healthcare technologies. By examining current research and proposing future directions for interdisciplinary collaboration, this work demonstrates how multiscale brain modeling can revolutionize both scientific understanding and clinical practice.

## 1 Introduction

The brain is an extraordinary organ, orchestrating dynamic processes that span multiple scales—from the molecular activity within neurons to the large-scale interactions between brain regions that govern thought, emotion, and behavior. Understanding this multiscale organization is essential for uncovering the fundamental principles of brain function and identifying the mechanisms behind its dysfunctions in neurological and psychiatric disorders (Jiang et al., 2024; Lu et al., 2022). Modern experimental advances, such as breakthroughs in genetics, molecular biology, cell physiology, and neuroimaging, have provided detailed insights into specific aspects of brain activity at each of these levels (Laasya et al., 2024; Yen et al., 2023). However, integrating these findings into a unified multiscale framework that bridges molecular, cellular, circuit, and systems-level dynamics remains one of the greatest challenges in neuroscience.

While significant progress has been made in isolating and characterizing brain processes at individual scales (Ozdemir et al., 2020; Poldrack et al., 2009), the brain’s complexity demands an approach that accounts for interactions across these levels. For instance, understanding how molecular disruptions, such as ion channel mutations, manifest as circuit-wide abnormalities or how these changes propagate to affect whole-brain dynamics and behavior requires sophisticated methods capable of capturing cross-scale relationships (Dulla et al., 2016; Kullmann, 2010). Addressing these challenges is not merely an academic exercise; it has profound implications for advancing our understanding of brain function in health and disease and for developing targeted interventions.

The emergence of advanced computational techniques, big data analytics, and informatics tools provides an unprecedented opportunity to construct multiscale models of brain function (Dura-Bernal et al., 2024). These models aim to integrate diverse datasets—ranging from genetic profiles and electrophysiological recordings to large-scale imaging data—into cohesive representations that can simulate interactions between neuronal populations and broader brain networks. By capturing these complex dynamics, multiscale models can offer insights into how microscopic phenomena drive macroscopic brain activity and behavior. Such approaches not only hold promise for unraveling the basic mechanisms of the brain but also for addressing critical questions in clinical neuroscience and applied fields such as neuroengineering and artificial intelligence.

This review explores the current state of multiscale brain modeling, examining the methodologies and tools that enable researchers to bridge the diverse scales of brain organization. It highlights key advances in experimental and computational techniques, as well as the challenges inherent in constructing accurate and predictive multiscale models. Furthermore, we discuss the potential applications of these models in clinical practice, neurological research, and the development of AI-based technologies. By synthesizing findings across disciplines and scales, multiscale brain modeling stands poised to unlock transformative insights into the brain’s workings, its pathologies, and its role in shaping human behavior and cognition.

## 2 Defining multiscale brain models

A multiscale brain model seeks to bridge the gap between microscopic (molecular, cellular) and macroscopic (whole brain, behavior) phenomena (Dura-Bernal et al., 2024). While we have models that simulate neuronal activity at the synaptic level, the challenge lies in scaling these simulations up to understand higher-order brain functions (Varley et al., 2023). In this section, we examine the methodologies used to achieve multiscale modeling and the key challenges inherent in this effort.

### 2.1 Microscopic scale: molecular and cellular models

At the microscopic scale (Deistler et al., 2024), advances in computational neuroscience have allowed researchers to model the biophysical properties of neurons and synapses in unprecedented detail.

Studies emphasize the importance of neurotransmitter dynamics, receptor interactions, and synaptic vesicle release mechanisms in shaping the overall network response. For instance, a study focused on asymmetric voltage attenuation along dendrites, shedding light on how ion channels and structural plasticity contribute to learning processes (Moldwin et al., 2023). Additionally, scientists provided quantitative insights into calcium-triggered neurotransmitter release, which plays a critical role in synaptic efficacy and homeostasis (Rizo, 2018).

Moreover, molecular-level models like the Hodgkin-Huxley framework simulate ion channel gating kinetics, offering a robust foundation for understanding action potential propagation (Kumar et al., 2024; Tekin, 2022). Innovations such as differentiable neural simulators have further extended traditional biophysical models by enabling the integration of large-scale transcriptomics and proteomics data to refine predictions about cellular responses in healthy and diseased states. Studies on voltage-gated calcium channels and their cooperative interactions have illustrated how subtle molecular changes can cascade into large-scale neuronal oscillations (Dave and Jha, 2021).

To ensure reproducibility and scalability, platforms such as Neuron and Blue Brain Project simulators have been employed to incorporate synapse-level data, enabling researchers to build more comprehensive, data-driven models of molecular signaling and network connectivity (Hjorth et al., 2021; Suzuki et al., 2012; Tretter, 2010).

These models have helped clarify how changes in neuronal behavior impact higher-order cognitive functions. However, while cellular-level models are valuable for understanding localized processes, connecting them to global brain activity remains an open question (Vila-Vidal et al., 2022).

### 2.2 Mesoscale: neuronal ensembles and microcircuits

Moving beyond individual neurons, models at the mesoscale level focus on microcircuits—localized networks of interconnected neurons that perform specialized tasks (Lee et al., 2023). Recent advances in connectomics and optogenetics have enabled detailed mapping of these circuits, which are thought to underlie core cognitive processes such as memory encoding and sensory processing (Berndt et al., 2023; Swanson et al., 2022). Network models, often employing graph theory, capture how information flows through these circuits (Anand A. et al., 2024; Sener et al., 2023). However, scaling up these models to understand how they interact across larger brain regions requires integrating additional layers of complexity, such as plasticity and feedback loops.

### 2.3 Macroscopic scale: large-scale brain networks

At the macroscopic level (Castaldo et al., 2022), computational models aim to describe large-scale brain networks that are responsible for coordinating sensory, motor, and cognitive functions (Pathak et al., 2022). These models often rely on data from non-invasive imaging techniques such as MRI, EEG, MEG, and PET, which offer ensemble measurements of brain activity. Functional connectivity analyses, which study the correlation patterns between different brain regions, provide valuable insights into network dynamics (Varga et al., 2024).

EEG and MEG, known for their high temporal resolution (Burgess, 2018), have been employed to model the timing and synchronization of neuronal populations. For instance, a study developed a multi-scale neural model using MEG data to simulate cortical excitability at the cellular level, providing insights into how fast oscillatory activity is modulated by synaptic interactions (van Nifterick et al., 2022). Similarly, another study demonstrated how EEG-informed computational models can simulate the propagation of alpha oscillations, linking macroscopic recordings to microscopic cellular dynamics (Sigala et al., 2014).

PET imaging, while traditionally associated with metabolic studies (Vizza et al., 2024), has also been used to create microscopic models of neurotransmitter activity. For example, scientists utilized PET-based kinetic modeling to investigate synaptic receptor binding and its contribution to neuron-to-neuron communication in microscopic circuits (Marner, 2012). This approach has been particularly valuable in studies of neurotransmitter dysfunction in psychiatric disorders, where synaptic changes often precede macroscopic brain alterations.

However, one of the most pressing challenges is inferring microscopic mechanisms (e.g., synaptic transmission) from such macroscopic data. The development of multiscale models could be pivotal in bridging this gap.

Emerging technologies are closing the gap between micro- and macroscales by integrating high-resolution molecular data with large-scale neuroimaging. Techniques such as two-photon microscopy and expansion microscopy provide subcellular resolution while capturing the architecture of larger neural circuits (Sneve and Piatkevich, 2022). For example, optogenetics and CRISPR-based neurogenetic tracing allow for precise manipulations of neuronal activity, enabling researchers to investigate causal relationships between molecular mechanisms and large-scale network dynamics (Seki et al., 2023).

When combined with neuroimaging techniques like MRI, fMRI, and MEG, these tools bridge the molecular, cellular, and system levels of brain analysis. For instance, studies have shown how transcriptomic profiles from the Allen Brain Atlas can be mapped onto large-scale connectomic data to better understand human brain network hubs’ vulnerability to neurodegenerative diseases (Anand C. et al., 2024; Xu et al., 2021; Xu et al., 2022).

By employing these integrative approaches, researchers can reveal the effects of genetic mutations and molecular dysregulation on whole-brain dynamics, facilitating more accurate modeling of disease progression and therapeutic interventions. These advancements highlight the potential of multi-modal tools in closing the gap between micro- and macroscales and enabling more comprehensive investigations into neural function across species and conditions.

### 2.4 Cross-species multiscale modeling

The study of neural dynamics across different species provides crucial insights into the evolutionary conservation and divergence of brain mechanisms, shedding light on the complexities of both normal cognitive functions and disease processes. Cross-species multiscale modeling integrates data at molecular, cellular, and system levels from animal models and humans, enabling researchers to make meaningful comparisons and generalizations (Kharche et al., 2022). However, this approach presents significant challenges alongside its potential benefits.

A major challenge lies in the comparability of datasets across species due to differences in anatomical structures, physiological processes, and experimental protocols. For instance, while the overall organization of brain regions may be conserved across species, there are substantial variations in cortical thickness, synaptic density, and neuronal firing patterns (Mahon, 2024). These differences can affect the interpretation of multiscale models and introduce inconsistencies in cross-species comparisons. Additionally, variations in data collection techniques—such as differences in temporal resolutions and imaging modalities—further complicate the integration of datasets from different species (Balk et al., 2022). Standardizing experimental conditions, developing interoperable data formats, and adopting common ontologies for neural components are essential steps toward improving data harmonization.

Numerous studies have demonstrated the importance of cross-species modeling in neuroscience research. For example, the Allen Brain Atlas has provided a comprehensive comparison of gene expression patterns across human and mouse brains, highlighting conserved and divergent pathways that influence brain function (Sunkin et al., 2013). Similarly, another study employed non-human primates to investigate neural regeneration, using their findings to inform human clinical trials for spinal cord injuries (Howard and Strittmatter, 2023).

Animal models, particularly rodents and non-human primates, provide a controlled environment for causal experimentation, allowing researchers to manipulate specific neural circuits and observe the resulting effects (Neziri et al., 2024). Optogenetic studies in rodents, for instance, have been pivotal in elucidating network-level dynamics that correspond to functional connectivity patterns observed in human fMRI studies (Moon et al., 2023). A study demonstrated how precise control of neuronal firing in mice could reproduce connectivity patterns seen in human imaging studies, bridging the gap between microcircuit dynamics and macroscopic observations (Ragone et al., 2023). These findings have been informed by studies by researchers who used combined EEG-fMRI approaches to validate similar network-level disruptions in neuropsychiatric conditions.

Emerging computational frameworks have further facilitated cross-species research by simulating disease mechanisms and testing hypotheses across biological scales. Machine learning algorithms and statistical modeling approaches can reconcile differences between species by accounting for anatomical and functional variations (Majumder and Mason, 2024). Comparative studies have also highlighted the importance of transcriptomic and proteomic data in linking molecular changes to large-scale brain network alterations (Weith et al., 2022).

Open-access repositories, supported by initiatives such as the Human Connectome Project and the BRAIN Initiative, have made significant strides in making cross-species datasets more accessible, thereby fostering collaborative research efforts (Lu et al., 2024). These platforms provide researchers with tools to integrate molecular data from animal models with human neuroimaging datasets, enabling comprehensive cross-species comparisons.

Cross-species multiscale modeling is instrumental in improving the translatability of preclinical findings to human applications (Shalash et al., 2024; Wang et al., 2022). By leveraging data from both animal and human studies, researchers can build more robust models that account for interspecies variability and refine predictions about disease progression and treatment outcomes. This approach holds promise for advancing precision medicine and developing more effective interventions for complex neurological disorders. Addressing the existing limitations in dataset compatibility and experimental design will further enhance the potential of cross-species multiscale modeling in neuroscience research.

## 3 Clinical implications of multiscale models

The potential clinical applications of multiscale brain modeling are vast. By providing a more accurate understanding of brain function and dysfunction, these models could transform diagnostic and therapeutic approaches for a range of neurological disorders. In this section, we discuss some of the most promising clinical applications of multiscale modeling.

### 3.1 Neurological disorders and personalized medicine

Multiscale models provide a powerful framework for uncovering the mechanisms underlying neurological disorders such as epilepsy, Alzheimer’s disease, and Parkinson’s disease, offering opportunities to advance both diagnosis and treatment. In epilepsy, for example, multiscale modeling enables the simulation of abnormal network activity progression, which can help pinpoint optimal intervention targets for therapeutic electrical stimulation (Yu et al., 2023). Studies have demonstrated that these models can identify specific brain regions or neural pathways where interventions like deep brain stimulation (DBS) can disrupt seizure propagation, improving treatment efficacy (Acerbo et al., 2022; Yang et al., 2023).

In the context of neurodegenerative diseases such as Alzheimer’s and Parkinson’s disease, multiscale models allow researchers to link cellular-level pathologies to their large-scale network effects. For instance, by simulating the accumulation and spread of amyloid-beta plaques in Alzheimer’s disease, these models provide insights into how molecular changes translate into cognitive deficits and network dysfunctions over time (Cabrera-Álvarez et al., 2023). Similarly, in Parkinson’s disease, multiscale approaches have been used to study how dopaminergic neuron loss impacts motor control circuits, aiding in the development of targeted therapies (Khan et al., 2023; Yan et al., 2024).

The integration of patient-specific data into multiscale models is particularly promising for advancing personalized medicine (Trezza et al., 2024). By incorporating individual variations in brain structure, connectivity, and functional dynamics, these models can predict how a specific patient might respond to various treatment options. For example, personalized simulations can optimize DBS parameters or predict the effectiveness of pharmacological treatments, tailoring interventions to a patient’s unique neural architecture (Sendi et al., 2024). This personalized approach not only enhances treatment outcomes but also minimizes side effects, aligning with the growing emphasis on precision medicine in healthcare.

### 3.2 Applications in brain-machine interfaces

Multiscale models hold significant promise for advancing brain-machine interfaces (BMIs), which rely on the precise decoding of brain signals to control external devices such as prosthetic limbs, communication tools, or robotic systems. BMIs translate neural activity into actionable commands, enabling individuals to interact with their environment despite severe physical limitations (Belwafi and Ghaffari, 2024). However, current BMI systems often face challenges such as signal variability, noise, and limited understanding of the multiscale dynamics underlying neural processes (Katoozian et al., 2024).

Multiscale brain models that integrate cellular, circuit-level, and network-level data could address these challenges by providing a more comprehensive representation of brain activity. At the cellular level, these models capture the intricate firing patterns and synaptic dynamics of neurons, while at the network level, they reflect the large-scale connectivity and dynamics of brain regions. Studies have shown that integrating data across these scales can enhance BMI performance by improving decoding algorithms and providing more robust control (Haghi et al., 2019; Hsieh et al., 2017; Li et al., 2022). For instance, researchers have emphasized the role of large-scale connectivity in improving motor control accuracy in BMIs designed for prosthetic limb use (Benz et al., 2012), while other studies have explored how synaptic-level details can reduce signal noise and variability (Gatys et al., 2015; Lotter et al., 2022).

The incorporation of multiscale insights into BMI design can significantly improve the accuracy and reliability of these systems. By modeling neural activity across multiple scales, BMIs can achieve finer control over external devices, enabling more natural and intuitive movements (Chen et al., 2020; Rouse and Schieber, 2015; Sorrell et al., 2021). This approach has proven particularly beneficial in clinical settings, such as restoring motor functions in patients with paralysis. Similarly, BMIs leveraging multiscale neural data have been applied in stroke rehabilitation, enabling targeted reactivation of neural circuits to support motor recovery (Jia et al., 2022).

Multiscale models also pave the way for personalized and adaptive BMI systems that account for individual differences in brain activity. By integrating data specific to a patient’s unique neural architecture and dynamics, these systems can adapt over time to changes such as neural plasticity, injury, or disease progression (Jia et al., 2022). Studies have demonstrated the potential of adaptive BMIs in managing neurodegenerative conditions like ALS, where neural patterns evolve as the disease progresses (Abbaspourazad et al., 2018; Fomina et al., 2016; Held et al., 2019). Personalized systems ensure long-term efficacy and usability, providing stable performance despite these changes.

Beyond restoring motor functions, multiscale BMIs have been explored in various other applications, such as enabling communication for individuals with locked-in syndrome or controlling robotic assistants in home care. Research has shown that integrating multiscale models into BMI systems can significantly improve their ability to interpret subtle neural signals, opening possibilities for broader applications in clinical and non-clinical settings (Hsieh et al., 2019; Kantawala et al., 2024).

In general, studies highlight that multiscale models offer a transformative approach to BMI development, addressing key limitations of existing systems and expanding their applicability. By bridging the gap between cellular-level neural activity and large-scale brain dynamics, these models enhance the precision, adaptability, and usability of BMIs, ultimately improving quality of life for patients and advancing the field of neurotechnology.

## 4 Technological impacts: AI and big data integration

One of the most exciting frontiers in multiscale brain modeling is its intersection with artificial intelligence (AI) and big data. These technologies can assist in the construction, validation, and scaling of brain models, while brain modeling, in turn, can inform the development of more efficient AI systems.

### 4.1 Enhancing AI algorithms

Artificial intelligence (AI) has made significant strides by leveraging biologically-inspired models such as artificial neural networks (ANNs) and convolutional neural networks (CNNs), which draw inspiration from the structure and function of the human brain (Schmidgall et al., 2024). However, current AI systems primarily rely on simplified abstractions of brain functions and often ignore the multiscale nature of neural computations that occur across cellular, circuit, and system levels (Badman et al., 2020).

Multiscale brain models in AI: The multiscale organization of the brain involves interactions at various levels, from molecular dynamics within neurons to the coordinated activity of large-scale networks such as the prefrontal cortex and hippocampus (Lewis et al., 2023). Studies highlight the significance of understanding the brain’s modular and hierarchical organization to achieve efficient information processing (Coward, 2024; Pathak et al., 2024). Incorporating such multiscale principles into AI could enable the development of systems that are both computationally efficient and capable of solving highly complex tasks in dynamic environments.

Efficiency and Robustness in Problem-Solving: The brain’s ability to solve problems efficiently is closely tied to its multiscale structure. For example, the interaction between local neural circuits and global brain networks facilitates both specialization and integration of information, allowing for adaptive and robust decision-making (Kar et al., 2012). By mimicking these principles, AI systems could exhibit enhanced adaptability and robustness. Autonomous systems, for instance, could benefit from these insights by improving their capacity to handle unstructured and unpredictable scenarios, such as those encountered in autonomous driving or robotic exploration.

Advancements in Machine Learning: Machine learning, a subset of AI, could greatly benefit from the incorporation of multiscale brain models. Current AI systems often face limitations such as high energy consumption and lack of generalization (Goetz et al., 2024). Recent studies have shown that energy-efficient computational models inspired by neural sparsity and hierarchical processing, such as spiking neural networks (SNNs), can achieve brain-like efficiency in specific tasks (Li et al., 2024; Wang et al., 2024). Extending these models with multiscale insights could further enhance their capability to generalize across tasks and reduce computational demands.

### 4.2 Big data and multiscale simulations

The advent of big data technologies has revolutionized neuroscience research, enabling the analysis of vast and diverse multimodal datasets such as genetics, neuroimaging, and electrophysiology (Chung et al., 2023). These large-scale datasets are pivotal in refining multiscale models of the brain, enhancing their accuracy, and providing unprecedented insights into the interactions between different levels of brain organization. By integrating data from multiple modalities, researchers can construct comprehensive models that bridge the molecular, cellular, circuit, and network levels, offering a holistic view of brain function and dysfunction.

### 4.3 Big data in multiscale modeling

Advances in data acquisition technologies, such as high-resolution MRI, single-cell RNA sequencing, and multi-electrode arrays, have significantly expanded the availability of multimodal brain data (Ramezani et al., 2023). Studies with the Human Connectome Project illustrate how integrating imaging, electrophysiological, and genetic data can create more detailed models of brain connectivity (Chung et al., 2023). Similarly, The Allen Brain Atlas has made significant contributions to brain mapping by providing comprehensive gene expression maps for both human and mouse brains. The atlas includes detailed data on spatial gene expression patterns and integrates transcriptomic data with anatomical and functional mapping. This has enabled researchers to link molecular-level processes to brain regions associated with specific functions or diseases (Martins et al., 2021).

The BigBrain atlas is a high-resolution, three-dimensional reconstruction of a human brain, created using serial histological sections at a resolution of 100 micrometers (Sainz Martinez et al., 2022). This project has provided an unprecedented level of detail in brain anatomy, allowing researchers to study the fine-grained cortical layers and their organization. By integrating this histological data with neuroimaging datasets, the BigBrain Project bridges the gap between cellular-level structures and whole-brain networks.

Brain Initiative Cell Census Network (BICCN) focuses on creating a complete cell-type atlas for the human, mouse, and non-human primate brains. By combining single-cell RNA sequencing with spatial transcriptomics, BICCN offers insights into the diversity of neural cell types and their roles in brain circuits (Jung and Kim, 2023). This multiscale dataset is crucial for understanding how different cell types contribute to the formation and function of brain networks.

Comparative projects, such as the Mouse Brain Atlas and the PRIME-DE (Primate Data Exchange), provide detailed brain maps across species (Milham et al., 2018; Zeng et al., 2015). These resources enable researchers to study conserved neural circuits and species-specific differences, aiding in the validation of preclinical findings and improving the translatability of animal model studies.

Initiatives like the UK Biobank and the Adolescent Brain Cognitive Development (ABCD) study have generated large-scale neuroimaging datasets from diverse populations (Bernhardt et al., 2023; Makowski et al., 2024). These open-access datasets include structural MRI, diffusion tensor imaging (DTI), and functional MRI data, facilitating cross-cohort comparisons and meta-analyses that enhance our understanding of brain structure-function relationships.

Machine learning has become indispensable in analyzing and processing these complex datasets. For example, studies demonstrated how machine learning can classify and map brain regions using multimodal data, greatly advancing our understanding of the brain’s functional and structural organization (Anbarasi et al., 2024; Luo et al., 2024). Deep learning techniques have also proven effective, with studies showing how biologically inspired neural networks outperform traditional methods in modeling sensory processing by predicting how neural activity propagates across scales (Hua et al., 2024).

Big data-trained multiscale models have enabled researchers to explore how various levels of brain organization interact under different conditions. For instance, researchers used data-driven modeling to simulate the impact of local synaptic changes on large-scale network dynamics (Marsh et al., 2024; Piccinini et al., 2022). These models not only improve accuracy but also offer actionable insights for therapeutic applications, such as brain stimulation techniques to treat neurological disorders.

### 4.4 The role of computing infrastructure

Analyzing and managing massive brain datasets requires cutting-edge computational power. Cloud computing and high-performance computing (HPC) systems play a critical role in storing, processing, and simulating these complex models. Platforms like EBRAINS, developed by the Human Brain Project, exemplify this synergy by integrating HPC and cloud-based tools to enable multiscale brain modeling. These resources allow researchers to perform simulations that would otherwise be impossible due to their computational intensity.

The demands of multiscale modeling are immense, particularly when working with datasets that include billions of variables, such as whole-brain transcriptomics or extensive neural recordings. As studies indicated, without sustained investment in HPC infrastructure and the optimization of algorithms, unraveling the brain’s multiscale organization will remain inaccessible to many research groups (Bastiani and Roebroeck, 2015; Bria et al., 2014).

Beyond enabling deeper exploration of brain function, enhanced computational capabilities are driving innovation in areas like drug discovery and personalized medicine (Lewandowski and Koller, 2023). Multiscale simulations, for example, have been instrumental in predicting how genetic mutations affect brain connectivity, paving the way for targeted therapies for conditions like epilepsy and schizophrenia (Lu et al., 2022; Lytton et al., 2017). These applications underscore the transformative potential of advanced computing in neuroscience and medicine.

## 5 Challenges and future directions

Expanding on multiscale brain modeling requires a comprehensive approach that considers both the current challenges and future directions within this emerging field. A key challenge lies in the need for extensive datasets that can capture brain activity across various scales. Current techniques such as electroencephalography (EEG), functional magnetic resonance imaging (fMRI), and magnetoencephalography (MEG) provide valuable insights but often lack the integration needed to connect cellular activity with large-scale network behaviors (Caznok Silveira et al., 2024). Research shows that combining multimodal data is essential for constructing accurate models that can bridge these scales (Gottipati and Thumbur, 2024; Luo et al., 2024). For example, advances in neuroimaging techniques are beginning to offer ways to connect micro-level data, such as gene expression, with macroscale brain activity, but more progress is required to make these methods scalable and clinically applicable (Hoang et al., 2024).

The computational complexity involved in modeling the brain at multiple scales is a significant obstacle that requires sophisticated solutions. The brain’s intricate neural architecture operates with highly non-linear dynamics, requiring simulations that capture both short-term processes, such as synaptic transmission and neural firing patterns, and long-term processes, including synaptic plasticity and memory consolidation. This complexity necessitates advanced algorithms and substantial computational power to ensure accurate and efficient modeling across spatial and temporal scales (Li et al., 2019).

Recent advancements in computational approaches have begun to address these challenges. Transformer-based neural networks, originally designed for natural language processing, have demonstrated considerable potential in approximating non-linear brain dynamics (Zhu et al., 2024). Their ability to handle sequential data efficiently allows for more accurate representations of neural interactions over time. Furthermore, neuromorphic computing systems—designed to mimic the structure and function of biological neurons—have emerged as a game-changer. These systems offer significant reductions in energy consumption and improvements in computational speed, making large-scale simulations more feasible (Raikar et al., 2024).

In addition to algorithmic innovations, the growth of cloud-based platforms and distributed computing infrastructures has played a crucial role in supporting brain modeling efforts. Cloud platforms provide scalable resources that can handle massive datasets, enabling researchers to perform real-time simulations and analyses of brain networks at multiple scales. Distributed computing allows the division of complex simulations across multiple nodes, reducing processing time and facilitating collaborative research across institutions (Liu and Zhao, 2022).

Despite these advancements, challenges remain, such as optimizing neuromorphic systems for broader applications and ensuring that distributed computing frameworks maintain data security and consistency. Future research must focus on enhancing the interoperability of these technologies and integrating them seamlessly into multiscale brain modeling workflows. By addressing these computational bottlenecks, researchers can make significant strides in understanding the brain’s function and dysfunction, leading to breakthroughs in neuroscience and the development of more precise diagnostic and therapeutic tools.

Acquiring datasets across scales remains a significant bottleneck due to the specialized equipment required for cellular and molecular imaging and the complexities of patient consent for clinical data collection. Integration requires harmonization across different data formats, temporal resolutions, and experimental methods. These challenges are compounded when datasets are collected across multiple institutions, often resulting in heterogeneity in protocols and data standards. For example, the BRAIN Initiative has aimed to overcome such hurdles by fostering technological innovations and collaborative frameworks that allow for large-scale neural data acquisition and sharing (Rahimzadeh et al., 2023). Similarly, the Human Cell Atlas initiative provides a roadmap for standardizing data collection and making comprehensive single-cell atlases publicly available, illustrating the power of open-access repositories in facilitating multiscale research (Kirby et al., 2024).

Recent advancements in machine learning and data integration techniques have enabled the combination of functional neuroimaging data (such as fMRI and EEG) with single-cell transcriptomics to generate more accurate multiscale representations of brain function. For instance, studies have demonstrated the utility of multi-modal approaches, where neuroimaging data are parcellated and combined with gene expression profiles to uncover the underlying cellular basis of observed neural activity patterns (Jiao et al., 2024). Platforms like the Human Connectome Project and frameworks for single-cell integration pipelines have highlighted the potential of these combined methodologies.

Moreover, machine-learning-driven frameworks enhance integration by addressing variations in temporal and spatial resolution. For example, integrating transcriptomic data with large-scale fMRI maps using computational models has helped resolve discrepancies in cross-scale analysis (Gryglewski et al., 2018; Selvaggi et al., 2021). These efforts underscore the potential of emerging technologies to bridge cellular, molecular, and functional scales in neuroscience research.

Interdisciplinary collaboration is critical to addressing the many challenges posed by multiscale brain modeling. Researchers from fields as diverse as neuroscience, data science, and computer engineering must come together to develop models that not only simulate brain activity but also translate into clinically relevant tools. For example, studies have shown that when clinicians work closely with data scientists, it leads to more precise models of neurological disorders, which could result in earlier diagnoses and more personalized treatments (Calderone et al., 2024; Manin et al., 2024).

Future research should also focus on integrating molecular, cellular, and network-level data into cohesive models that offer a holistic view of brain function. This integration is vital for answering key questions about how microscopic cellular events contribute to large-scale phenomena such as behavior and cognition. For example, recent studies have attempted to model how disruptions at the cellular level, such as protein misfolding, lead to neurodegenerative diseases like Alzheimer’s (Patel et al., 2024; Ramazi et al., 2024). However, the field is still far from a comprehensive model that integrates these disparate scales of brain function.

Moreover, ensuring the clinical validity of multiscale models is paramount. Without rigorous validation against clinical outcomes, the insights provided by these models may not translate into actionable strategies for diagnosis and treatment. One promising avenue is using patient-specific data to create individualized brain models that can predict how a patient’s condition will progress, thus enabling more targeted treatments. Clinical trials and collaborations with healthcare providers are needed to establish the real-world utility of these models.

Finally, as multiscale brain modeling increasingly intersects with artificial intelligence (AI) and brain-machine interfaces, ethical considerations will become even more important. Questions surrounding data privacy, the role of AI in clinical decision-making, and the societal impact of such technologies must be addressed. As these models begin to shape both research and clinical practices, establishing ethical frameworks will be essential to ensuring they are used responsibly and equitably.

## 6 Conclusion

Multiscale brain modeling is an innovative and rapidly advancing field that holds immense promise for transforming our understanding of the brain’s complex structure and function. These models aim to bridge the gap between the microscopic and macroscopic levels of brain organization, connecting cellular and molecular processes to the larger-scale dynamics observed in brain networks and behavior. By capturing interactions across these scales, multiscale models offer a more comprehensive view of how the brain functions under normal conditions and how it undergoes changes in pathological states.

One of the key strengths of multiscale brain modeling lies in its ability to integrate diverse types of data, ranging from molecular imaging and electrophysiological recordings to whole-brain imaging techniques like fMRI and MEG. This integration enables researchers to develop more accurate and predictive models of brain activity, shedding light on phenomena that are difficult to understand when studied at a single scale. For instance, multiscale models can help identify how microscopic disruptions, such as ion channel dysfunctions or synaptic abnormalities, lead to macroscopic manifestations like epileptic seizures or neurodegenerative disorders.

The potential applications of multiscale brain modeling extend far beyond basic neuroscience. Clinically, these models could revolutionize how we diagnose and treat neurological and psychiatric disorders. By simulating brain activity across scales, researchers can develop personalized treatment strategies, such as optimizing stimulation protocols for deep brain stimulation or tailoring drug interventions based on individual brain dynamics. In the technological domain, multiscale brain models could inspire advances in brain-computer interfaces (BCIs), artificial intelligence (AI), and neuroprosthetics, driving the development of systems that mimic or augment brain functions with unprecedented accuracy.

To fully realize the potential of multiscale brain modeling, continued advancements in several key areas are essential. First, computational methods must evolve to handle the immense complexity of multiscale data, requiring new algorithms, machine learning techniques, and high-performance computing resources. Second, progress in data acquisition technologies, such as high-resolution imaging and multimodal data integration, will be critical to provide the detailed inputs needed for these models. Finally, fostering interdisciplinary collaboration between neuroscientists, engineers, mathematicians, and clinicians will be vital for translating theoretical models into practical tools that can address real-world challenges.

As the field continues to grow, multiscale brain modeling is poised to unlock transformative insights into the brain’s workings and its disorders, providing a foundation for breakthroughs in neuroscience, medicine, and technology. By bridging scales and integrating knowledge across disciplines, these models represent a powerful approach to addressing critical questions about the brain and its role in shaping human behavior and health.
